# Supplementary material for: Broad H3K4me3 domains support oocyte genome silencing and maturation but are dispensable for repression in early embryos
Source: Development. 2025 Oct 27;152(21):dev204638. doi: 10.1242/dev.204638 (PMC12633791; doi:10.1242/dev.204638)
Supplement: Supplementary information [file develop-152-204638-s1.pdf]

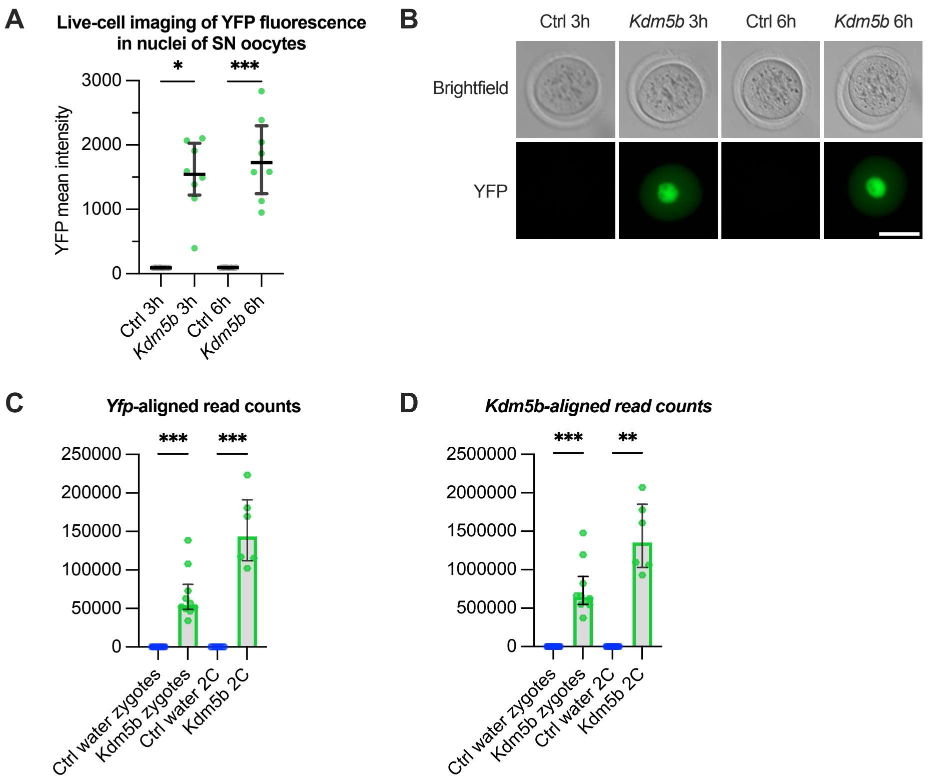

Fig. S1. Validation of KDM5B overexpression by YFP fluorescence and RNA-seq.
(A) YFP fluorescence intensity in nuclei of SN oocytes following live-cell imaging at 3 h and 6 h post-injection, comparing uninjected controls (Ctrl) and *Kdm5b-yfp* mRNA-injected oocytes (*Kdm5b*). Total number of oocytes (one biological replicate): Ctrl n = 8 and *Kdm5b* n = 8. Lines and error bars: median and IQR. Pre-selected pairs were compared using the Friedman test (repeated measures design) with Dunn's multiple comparisons test; adjusted significance indicated by P value notation.
(B) Representative live-cell microscopy images of SN oocytes corresponding to (A). Images show brightfield and YFP fluorescence, with brightness and contrast adjusted consistently across samples (scale bar: 50 µm).
(C) *Yfp*-aligned read counts from the RNA-seq dataset described in Fig. 4A for all samples: Ctrl water zygotes (n = 10), *Kdm5b* zygotes (n = 10), Ctrl water 2C (n = 9), and *Kdm5b* 2C (n = 6). Bars and error bars: median and IQR. Pre-selected pairs were compared using Kruskal–Wallis with Dunn's multiple comparisons test; adjusted significance indicated by P value notation. Read counts were obtained by Bowtie2 paired-end alignment to the YFP coding sequence (from pcDNA3-YFP; see Materials and Methods), followed by quantification with featureCounts.
(D) *Kdm5b*-aligned read counts from the RNA-seq dataset as in (C). Bars and error bars: median and IQR. Pre-selected pairs were compared using Kruskal–Wallis with Dunn's multiple comparisons test; adjusted significance indicated by P value notation. Read counts were extracted from the existing gene count table (see Materials and Methods).
P value notation: ns $P > 0.05$, * $P \leq 0.05$, ** $P \leq 0.01$, *** $P \leq 0.001$.

Development • Supplementary information

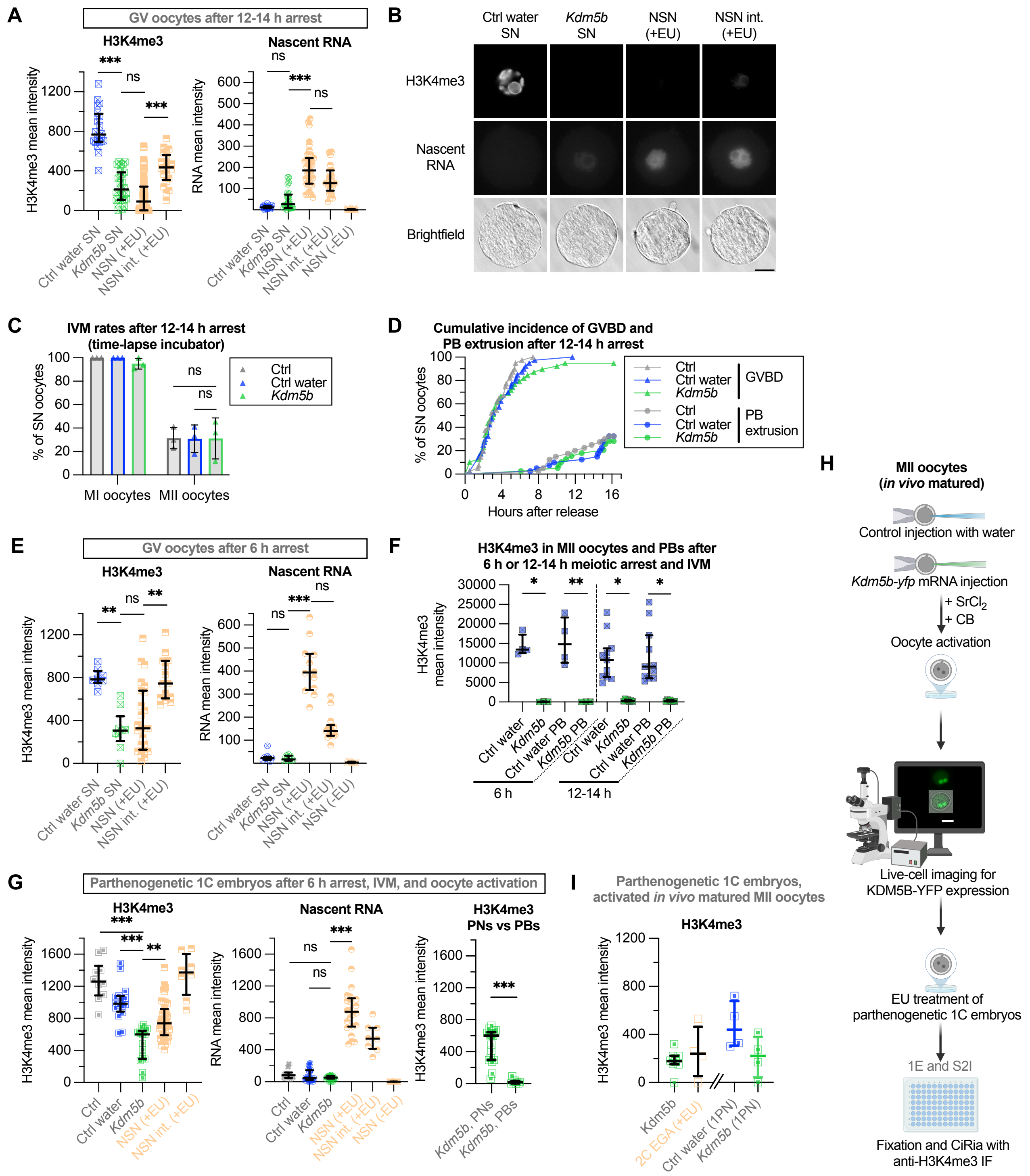

Fig. S2. Additional results following 12-14 h and 6 h meiotic arrest after injection of GV oocytes, as well as injection of *in vivo* matured MII oocytes, associated with Fig.1.

(A) H3K4me3 and nascent RNA signal intensity in nuclei of injected SN-stage GV oocytes, with NSN and intermediate NSN-SN (NSN int.) oocytes for comparison, all following 12–14 h meiotic arrest and 2 h EU labelling. Total number of oocytes (summed across two biological replicates): Ctrl water SN n = 20, *Kdm5b* SN n = 27, H3K4me3 NSN (+EU) n = 57, NSN int. (+EU) n = 21, RNA NSN (+EU) n = 39, and NSN (-EU) n = 22. Lines and error bars: median and IQR. Pre-selected pairs were compared using Kruskal–Wallis with Dunn's multiple comparisons test; adjusted significance indicated by P value notation.

(B) Representative microscopy images of GV oocytes corresponding to (A). Panel shows H3K4me3 and nascent RNA fluorescence together with brightfield images. Brightness and contrast were adjusted consistently across all samples (scale bar: 20 µm).
(C) Maturation after 15.1–16.2 h IVM following 12–14 h meiotic arrest. Each triangle represents one biological replicate. Bars and error bars: mean ± s.d., showing mean percentage reaching MI and MII stages. Total number of oocytes (summed across three replicates): Ctrl SNs n = 40, Ctrl water SNs n = 40, and *Kdm5b* SNs n = 39. Pairwise comparisons by Fisher's exact test on pooled counts; significance indicated by P value notation.
(D) Cumulative incidence plot of GVBD and PB extrusion over time following 12–14 h meiotic arrest. SN oocytes from start are the same as in (C). Total number of oocytes (summed across three biological replicates): Ctrl MI/MII n = 40/13, Ctrl water MI/MII n = 40/13, and *Kdm5b* MI/MII n = 37/11. Median time to GVBD: Ctrl = 2.9 h, Ctrl water = 3.1 h, and *Kdm5b* = 3.1 h; median time to PB extrusion: undefined in all groups (did not reach 50%). No statistically significant differences were found using pairwise log-rank (Mantel–Cox) tests with Holm–Šídák correction for multiple comparisons (alpha = 0.05); no P value notation indicated.
(E) H3K4me3 and nascent RNA signal intensity in nuclei of injected SN-stage GV oocytes and in NSN or intermediate NSN-SN (NSN int.) oocytes for comparison, all following 6 h meiotic arrest and 2 h EU labelling. Number of oocytes (one replicate): Ctrl water SN n = 8, *Kdm5b* SN n = 9, H3K4me3 NSN (+EU) n = 24, NSN int. (+EU) n = 14, RNA NSN (+EU) n = 12, and NSN (-EU) n = 12. Lines and error bars: median and IQR. Pre-selected pairs were compared using Kruskal–Wallis with Dunn's multiple comparisons test; adjusted significance indicated by P value notation.
(F) H3K4me3 signal intensity in chromatin of MII oocytes and PBs following 6 h or 12–14 h meiotic arrest post-injection at GV SN-stage and 15.8–16.8 h IVM. Total number of oocytes: 6 h Ctrl water MII and PB n = 4 and *Kdm5b* MII and PB n = 3 (one replicate); 12–14 h Ctrl water MII and PB n = 11 and *Kdm5b* MII and PB n = 7 (summed across two replicates). Unlike other signal intensity data, values here were obtained by confocal microscopy. Lines and error bars: median and IQR. Pre-selected pairs were compared using Kruskal–Wallis test with Dunn's multiple comparisons test; adjusted significance indicated by P value notation.
(G) Left and middle: H3K4me3 and nascent RNA signal intensity in parthenogenetic 1C embryos after chemical activation following 6 h meiotic arrest post-injection at GV SN-stage and 15–16 h IVM, with NSN and intermediate NSN-SN (NSN int.) oocytes for comparison. Total number of samples (summed across two biological replicates): Ctrl n = 5, Ctrl water n = 8, *Kdm5b* n = 9, H3K4me3 NSN (+EU) n = 40, NSN int. (+EU) n = 8, RNA NSN (+EU) n = 22, and NSN (-EU) n = 18. Parthenogenetic 1C embryos were EU-labelled for 4 h, fixed 11 h after activation, and stained. Lines and error bars: median and IQR. Pre-selected pairs were compared using Kruskal–Wallis test with Dunn's multiple comparisons test; adjusted significance indicated by P value notation.
Right: H3K4me3 signal intensity in pronuclei (PN) and polar body (PB) chromatin of parthenogenetic 1C embryos in the *Kdm5b* group (subset of data in left graph). Lines and error bars: median and IQR. Pre-selected pair was compared using Mann–Whitney test; significance indicated by P value notation.
(H) Experimental workflow for injection and chemical activation of *in vivo*-matured MII oocytes. Oocytes were injected with *Kdm5b-yfp* mRNA directly after isolation; water-injected oocytes served as controls. Next, they were activated within 20 min post-injection by strontium chloride ($SrCl_2$) and cytochalasin B (CB). The C-terminal YFP tag enabled live-cell confirmation of KDM5B expression (scale bar: 50 µm). Parthenogenetic 1C embryos were EU-labelled for 2 h, fixed 9–10 h after activation, and processed for Click-iT RNA imaging assay (CiRia) with anti-H3K4me3 IF. Created in BioRender. Indahl,

Development • Supplementary information

M. (2025) https://biorender.com/zq6cibi

(I) H3K4me3 signal intensity in parthenogenetic 1C embryos following chemical activation after injection at MII stage (*in vivo* matured), with 2C embryos at mid-EGA (orange) as reference. Number of samples (corresponds to the first replicate in Fig. 1E): *Kdm5b* n = 4, 2C EGA (+EU) n = 2, Ctrl water (1PN) n = 4, and *Kdm5b* (1PN) n = 4. No parthenogenetic 1C embryos with two PNs were obtained in the Ctrl water group in this replicate; therefore, the 1PN groups are included (plotted to the right of the broken x-axis). H3K4me3 values from the 1PN groups were used to calculate the ratio shown in the second bar of Fig. 3E (left). Lines and error bars: median and IQR. No statistical analysis due to small sample size.

P value notation: ns $P > 0.05$, * $P \leq 0.05$, ** $P \leq 0.01$, *** $P \leq 0.001$.

Development • Supplementary information

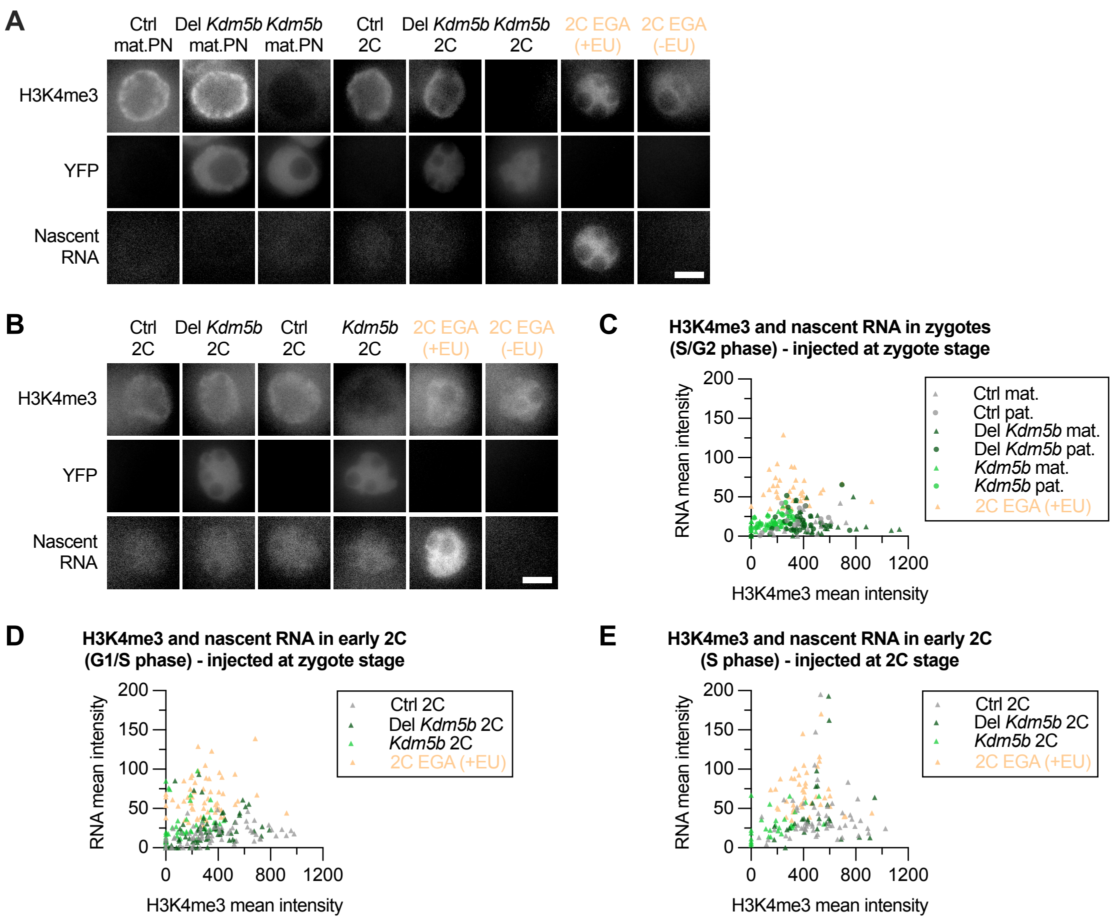

Fig. S3. H3K4me3 and nascent RNA signal intensities from Click-iT RNA imaging assay (CiRia) with anti-H3K4me3 IF staining, associated with Fig.3.

(A) Representative fluorescence microscopy images of zygotes (mat. PN = maternal pronucleus) and 2C embryos corresponding to Fig. 3B and 3C. Panel shows H3K4me3, YFP, and nascent RNA after background subtraction, with brightness and contrast adjusted consistently across all images (scale bar: 10 µm).

(B) Representative fluorescence microscopy images of 2C embryos corresponding to Fig. 3D. Panel shows H3K4me3, YFP, and nascent RNA after background subtraction, with brightness and contrast adjusted consistently across all images (scale bar: 10 µm).

(C) H3K4me3 signal intensity (x-axis) plotted against nascent RNA signal intensity (y-axis), corresponding to Fig. 3B.

(D) H3K4me3 signal intensity (x-axis) plotted against nascent RNA signal intensity (y-axis), corresponding to in Fig. 3C.

(E) H3K4me3 signal intensity (x-axis) plotted against nascent RNA signal intensity (y-axis), corresponding to Fig. 3D.

Development • Supplementary information

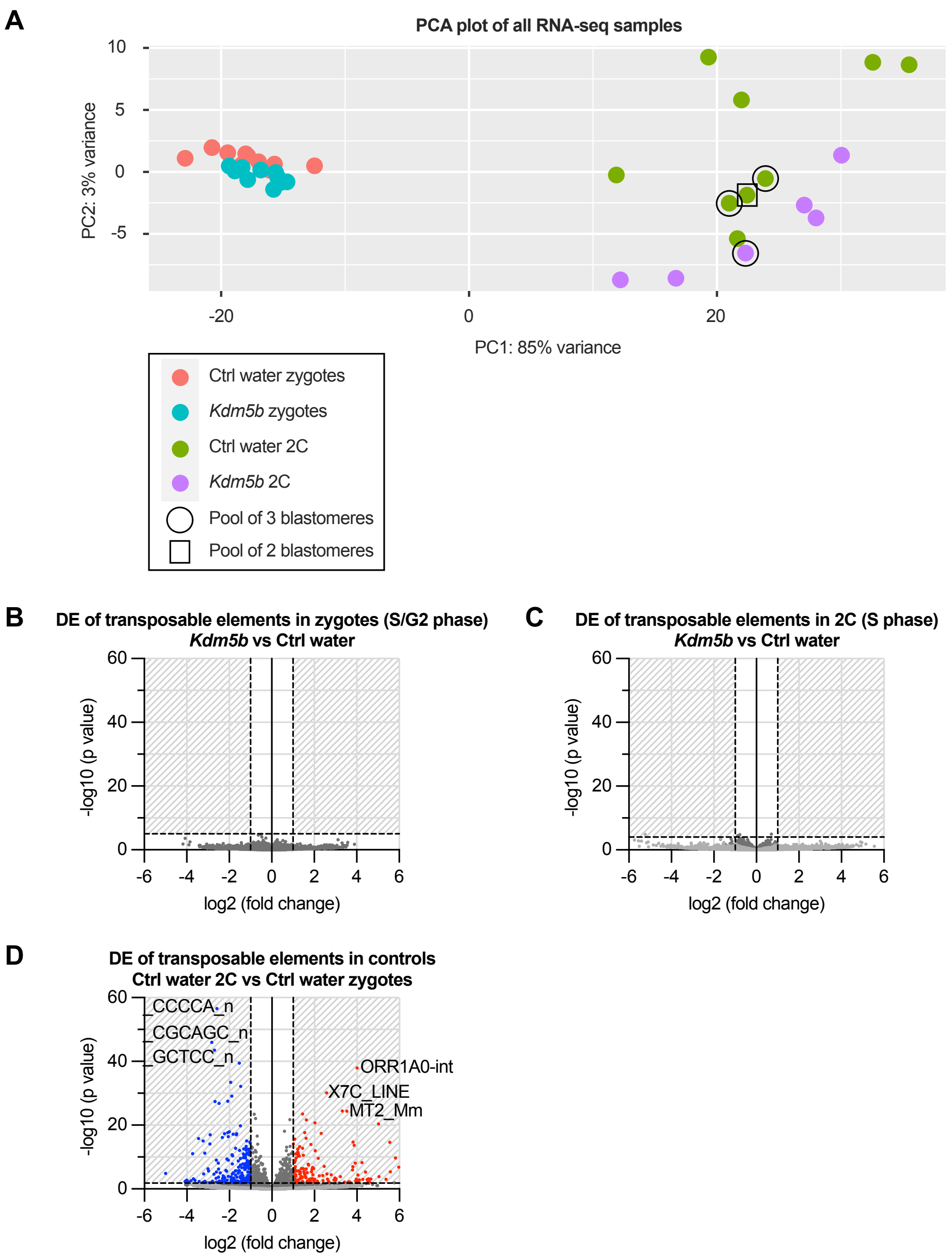

Fig. S4. Further analyses of RNA-seq data obtained as described in Fig. 4A.

(A) Principal component analysis (PCA) of RNA-seq data from all samples: Ctrl water zygotes (n = 10), *Kdm5b* zygotes (n = 10), Ctrl water 2C (n = 9), and *Kdm5b* 2C (n = 6). Each dot represents one sample. Pooled 2C samples consisting of three blastomeres from different embryos are encircled; the pool of two blastomeres is marked by a square. Samples without markings represent single zygotes or single 2C embryos with both blastomeres intact (see Materials and Methods for details).

(B) RNA-seq volcano plot showing DE of transposable elements in *Kdm5b*-injected zygotes (n = 10) versus Ctrl water zygotes (n=10).

(C) RNA-seq volcano plot showing DE of transposable elements in *Kdm5b*-mRNA injected 2C (n = 6) versus Ctrl water 2C (n = 9).

(D) RNA-seq volcano plot showing DE of transposable elements in Ctrl water 2C (n = 9) versus Ctrl water zygotes (n = 10). The three most significantly up- and downregulated elements are labelled.

Development • Supplementary information

For all volcano plots: SalmonTE was used to quanEfy transposable elements prior to DE analysis (see Materials and methods). Transposable elements with adjusted P < 0.05 and log2(fold change) > 1 or < -1 are shown as red (upregulated) or blue (downregulated) points. All coloured points fall within the shaded region denoting the significance threshold. Points in lighter grey indicate elements with undefined adjusted P values due to low mean normalised counts.

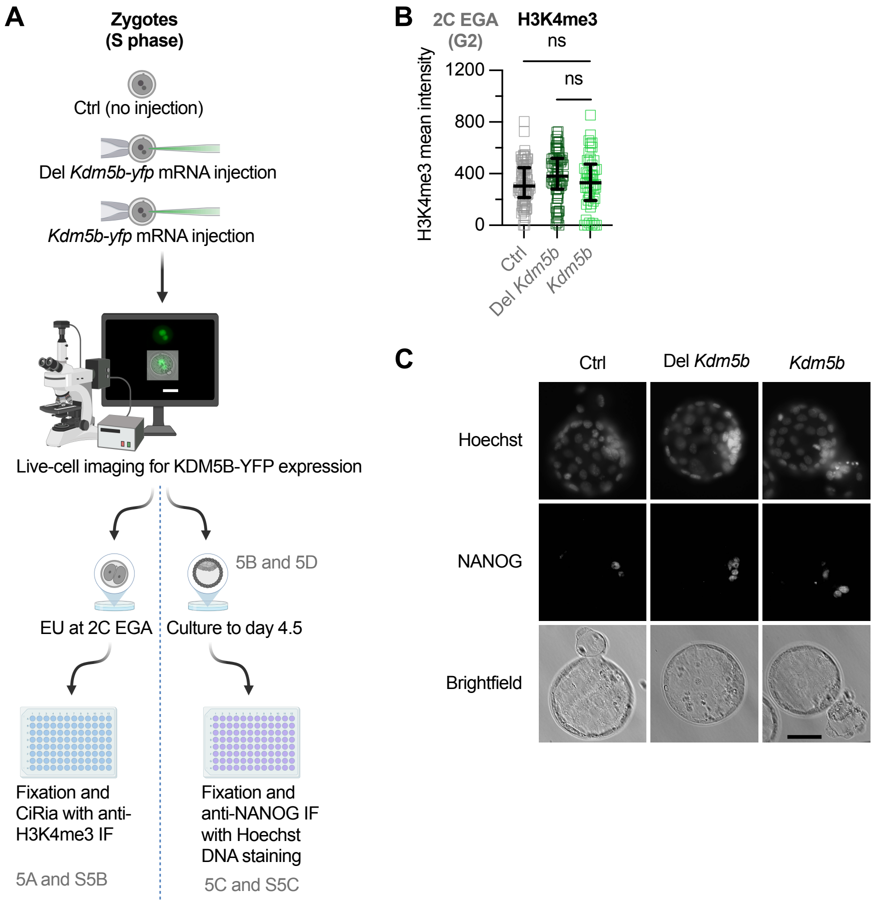

Fig. S5. Experimental workflow and additional data corresponding to Fig. 5.

(A) Experimental workflow: S-phase zygotes were injected with *Kdm5b-yfp* mRNA; uninjected zygotes and zygotes injected with mRNA encoding a catalytically inactive KDM5B (Del *Kdm5b-yfp*) served as controls. The C-terminal YFP tag enabled live-cell confirmation of KDM5B expression (scale bar: 50 µm). Zygotes were either (1) cultured to the 2C stage, EU-labelled for 2 h, fixed on average 15 h after division (range: 10–20 h, ~20–23 h post-injection), and processed for Click-iT RNA imaging assay (CiRia) with anti-H3K4me3 IF, or (2) cultured to developmental day 4.5, fixed on average 94 h post-injection (range: 93–95 h), and stained with anti-NANOG IF and Hoechst 33342 to enable cell counting in blastocysts. Created in BioRender. Indahl, M. (2025) https://biorender.com/ft2n82f

(B) H3K4me3 signal intensity in nuclei of 2C embryos at mid-EGA following injection at zygote stage. Total number of samples (summed across three biological replicates): Ctrl 2C n = 36.5, Del *Kdm5b* 2C n = 35, and *Kdm5b* 2C n = 25. Lines and error bars: median and IQR. Pre-selected pairs were compared using Kruskal–Wallis test with Dunn's multiple comparisons test; adjusted significance indicated by P value notation.

(C) Representative microscopy images of day 4.5 blastocysts corresponding to data in Fig. 5C. Panel shows DNA (Hoechst 33342) and NANOG fluorescence together with brightfield images. Brightness and contrast were adjusted consistently across samples (scale bar: 50 µm).

P value notation: ns P > 0.05, * P ≤ 0.05, ** P ≤ 0.01, *** P ≤ 0.001.
